# Ethnic differences in medicinal plant use among University students: a cross-sectional survey of self-reported medicinal plant use at two Midwest Universities

**DOI:** 10.1186/s12906-015-0725-1

**Published:** 2015-06-23

**Authors:** Rachel Craft, Katrina C. McClure, Steven Corbett, Maria Pontes Ferreira, Ashley M. Stiffarm, Kelly Kindscher

**Affiliations:** Department of Sociology, University of Kansas, 1415 Jayhawk Blvd., 730 Fraser Hall, Lawrence, KS 66045 USA; Department of Geography, University of Kansas, 1475 Jayhawk Blvd., 213 Lindley Hall, Lawrence, KS 66045 USA; Kansas Biological Survey, University of Kansas, 2101 Constant Ave., Lawrence, KS 66047 USA; Department of Nutrition & Food Service, College of Liberal Arts & Sciences, Wayne State University, Detroit, MI 48202 USA; Department of Environmental Science, Haskell Indian Nations University, Sequoyah 140, 155 Indian Avenue, Lawrence, KS 66046 USA

**Keywords:** US, Alternative medicine, Herbal medicine, Native American, Health disparities, Student health, College students, Surveys, Undergraduates

## Abstract

**Background:**

Numerous surveys of medicinal plant use among college students abound, but none compare use between students enrolled in two different Universities with significantly different ethnic compositions. The objective of this study is to compare medicinal plant use between two different ethnic college populations and explore differences between student medicinal plant users and non-users for comparison with previous research.

**Methods:**

Students (n = 721) at a large research university (n = 498) and a Pan-Tribal University for Native Americans (n = 233) completed surveys in October 2011 to assess past year medicinal plant use. The Mann-Whitney *U* test, Chi Square test, and General Linear Model were used to compare demographics and self-reported use of medicinal plants among students at both Universities and between past year users and non-users.

**Results:**

Over 23 % of university students surveyed reported past year medicinal plant use. Users were more likely to use commercial tobacco products and to report poorer health than non-users. While Native American student medicinal plant users reported significantly higher rates of commercial tobacco use, lower self-assessment of health, and less use of prescription medicine than non-Native users, no significant differences in prevalence of medicinal plant use were found between University student populations.

**Conclusions:**

Results are consistent with preexisting data showing higher rates of medicinal plant use among college students compared to the larger US population of adults and demonstrate previously documented health disparities in Native American populations compared to non-Native Americans.

**Electronic supplementary material:**

The online version of this article (doi:10.1186/s12906-015-0725-1) contains supplementary material, which is available to authorized users.

## Background

Adult use of dietary supplements in the US has increased significantly in recent years. While in 1990 only 2.5 % of the US adult population used a dietary supplement in the past year, use of non-vitamin, non-mineral dietary supplements increased over seven-fold to 17.9 % in 2012 [1–5]. Scholars believe this increase in use occurred because dietary supplements became more widely available due to economic forces, political factors, and the increasing availability of information online [6–10]. Economic factors include the increase of: insurance coverage of Complementary and Alternative Medicine (CAM), pharmaceutical manufacturing of CAM products, increased CAM advertising, and dietary supplement availability in superstores. Political factors include increased licensing of CAM professionals, the 1994 passage of the Dietary Supplement Health and Education Action, the creation of the National Center for Complementary and Alternative Medicine (changed to the National Center for Complementary and Integrative Health in December 2014), and increased social movement support in the form of women, gay, elderly, disabled, and other health-based rights movements.

### Previous research

Numerous surveys characterize herbal supplement use, a subset of dietary supplements that does not include vitamins, minerals, and other natural products that are not wholly derived from plants. Typical herbal supplement users in the US are middle-age, middle-class, college educated, white, and female [1–3, 5, 11–13]. Some surveys indicate that users self-rate their health as worse than non-users [12, 14], while others indicate that herbal supplement users report better health status [15, 16]. Users also exercise more than non-users [11]. Barnes et al found that the use of herbal supplements in the US ranges from 24.4 % in the West, to 20.4 % in the Midwest, 18.2 % in the Northeast, and 17.7 % in the South [1]. Data generated by Gardiner et al, Peregoy et al, and Kennedy indicate similar regional use patterns [3, 5, 11].

The most commonly cited sources of information for herbal supplement use are friends and family, media, and health stores [15, 17–19]. The most often used herbal supplements are Echinacea, ginseng, Ginkgo, garlic, St. John’s wort, Peppermint, ginger, and kava kava [1, 3, 11]. The top reasons cited for using herbal supplements are to improve health or well-being, relieve symptoms associated with chronic illness, prevent chronic disease, and to supplement a poor diet or unhealthy lifestyle [1, 11, 13].

Studies have also been performed on population subgroups in the US, including: hospitalized patients [12, 18], psychiatric outpatients [20], adults with depression [21], patients with cardiovascular disease [22], pregnant women with midwives [23], older adults [24], caregivers of children who use WIC [19], and Hispanic college students [25].

Studies on US college students indicate a greater prevalence of herbal supplement use with a similar demographic profile to the larger US adult user population (i.e., white, female). This makes college students an ideal demographic to explore the variation of herbal supplement use. The percent of college students who used an herbal supplement in the past year range from 79 % of students surveyed at an unnamed large state university [26], to 54 % of undergraduates surveyed at a midsize Midwestern university [27], 51 % of surveyed Rutgers University students [15], 48.5 % of surveyed Washington State University students [28], and 26.3 % of surveyed students at an urban, mid-sized university [17]. Of these college student surveys, the most commonly used herbal supplements are Echinacea, ginseng, St. John’s wort, chamomile, aloe, and ginger, which is comparable to US adult use studies. The most common indications for students’ use are to promote health, prevent illness (particularly physical), treat depression and anxiety [29], and lose weight.

Despite some evidence that college students have a higher prevalence of herbal supplement use than the rest of the American adult population, CAM and herbal supplement use in college students remains understudied [27]. By way of the 1994 Dietary Supplement Health and Education Act, herbal products are considered dietary supplements that can be bought and sold without demonstrating evidence of their safety or efficacy. Given the difficulty of ascertaining the safety of herbal supplement use, as demonstrated by contradictory and incomplete results that characterize much of the research in this vein, and the possibility of herb-drug interactions, many scholars indicate a need for research on the use and safety of herbal supplements [30–32].

### Research hypotheses

The purpose of this study was to establish the medicinal plant and herbal product usage frequency and characteristics of users among US college students in 2011. We chose to limit our inquiry to the use of medicinal plants, in order to distinguish the use of plants for medicine from other dietary supplements and to remain consistent with the focus of the University of Kansas Native Medicinal Plant Research Program. Lawrence, Kansas is home for two universities: The University of Kansas and Haskell Indian Nations University. The University of Kansas is a public research university and the largest university in Kansas, with an enrollment of nearly 30,000 students. Haskell Indian Nations University and Southwestern Indian Polytechnic Institute in Albuquerque, New Mexico are the only two US universities for Native Americans that are federally operated and funded by the Bureau of Indian Education. Haskell Indian Nations University offers Associate and Baccalaureate degrees, and has an enrollment of approximately 800 students (nearly twice that at the Southwestern Indian Polytechnic Institute). The proximity of these two schools in the same community allows for the expansion of the analysis to include a more in-depth investigation of ethnic differences in herbal product use.

Our hypothesis was that the Native American students from Haskell Indian Nations University (HINU) would utilize medicinal plant and herbal products more often than non-Native students from the University of Kansas (KU), assuming a greater cultural exposure to traditional plant medicine and fewer financial resources for pharmaceuticals [33]. Previous studies indicate that those who identify more intensely with being Native American and are involved in traditional Native American spirituality are more likely to embrace CAM practices [34, 35]. Fuchs and Bashshur demonstrated that strong cultural ties to a Native American community can predict traditional Native American medicinal plant use [36]. In a review of literature, Johnston found that CAM use by Native Americans is dependent upon the intensity of assimilative forces affecting cultural traditions and the degree of cultural resurgence following the marginalization of cultural practices [37]. Therefore we explored the differences between HINU and KU student users to determine if there would be any variation in use rates and patterns. This is the first student survey designed to focus on medicinal plant use and to compare use between two different universities in the same US Midwestern city that feature significantly different ethnic demographics.

Additionally, consistent with previous student survey work (discussed above), we also wanted to contribute data on the rate of medicinal plant use among these student populations and to explore differences between student user and non-user groups. To this end we report overall use rates and patterns of use among these student populations for comparison with preexisting research on herbal use among college student populations. Additionally, we compare demographic, self-health assessment, and use of tobacco products between student user and non-user groups, again for comparison with preexisting research.

## Methods

The HINU/KU student cross-sectional survey was drafted with similar questions used in previous college student surveys so as to allow for comparisons across time and space with preexisting research on the rate of college student use of medicinal plants and herbal supplements [15, 17, 26, 28, 38]. Internal Review Board approvals were granted at both KU and HINU. Surveys were administered to students during classes in the Fall 2011 semester at both universities. Courses were selected for survey distribution to maximize the diversity of students responding and allow for comparable student populations between both Universities. At KU, a sociology course that satisfies the social science requirement for Liberal Arts and Sciences majors at KU was selected for survey distribution. And at HINU, Freshman English and psychology courses that satisfy the General Education requirement for all students were surveyed at HINU. An author at HINU, and an author or Graduate Teaching Assistant at KU explained the purpose of the survey and read an oral consent statement to each class prior to administering the survey during class time. Surveys were completed within 5-10 min at the beginning or end of class and collected by the researcher or instructor. Students were given the option of not participating. No students at HINU chose not to participate, while less than 10 blank surveys were returned by KU students.

Part one of the survey (Additional file 1) included demographic variables (ethnicity, age, grade level, and gender), self-evaluation of health (on a five point likert scale ranging from good to poor), and health behavior questions. Part two of the survey consisted of medicinal plant and herbal product use questions, beginning with lifetime and past year use. If the student indicated lifetime use, they were asked to indicate their age at first use, from where or whom they learned how to use, how much money they spent on medicinal plants and herbal products per month, and whether they informed their physician about their use. All survey respondents were asked whether their physician asked them about medicinal plant or herbal product use, whether they take over-the-counter or physician-prescribed medication, and if they found sufficient information (from any source) on the use of medicinal plants and herbal products. Students who indicated past year use were additionally asked in Part three of the survey to review a list of commonly used medicinal plants and to specify the frequency and duration of use, the form of the plant used (e.g., dried, fresh, tincture, capsule/pill, topically, or infusion), reasons for use, and whether the plant was effective or had adverse effects. Although students were given the opportunity to add medicinal plants not already listed on the survey, we did not aim to survey all traditional medicinal plant use, nor were we seeking information on the spiritual use of plants. We anticipated that some HINU students may be reluctant to provide this information in order to protect sacred knowledge or cultural traditions.

Data were entered into a Microsoft Excel spreadsheet. Parts one and two survey data were coded into nominal and interval variables and imported into IBM SPSS Statistics 20. All survey data were descriptively analyzed in Excel; while SPSS was additionally used to statistically analyze Part one and two survey data. The Mann-Whitney *U* test and Chi Square test were used to compare demographics, health behaviors and medicinal plant use rates and characteristics between KU and HINU, and to explore differences between past year users and non-users in both student populations. A General Linear Model (GLM) was used to identify which of the many variables surveyed demonstrated the most variation in medicinal plant use patterns. GLM analysis was used only for those participants identified as users who completed Part three of the survey. Part two question answers, “Not Applicable or Don’t Remember” and “Haven’t looked for information,” were omitted from statistical analysis.

Reliability of survey responses regarding the use of medicinal plants were ascertained by comparing the consistency of answers to question 1 (“Have you ever used medicinal plants or herbal products for health or well-being”) and question 2 (“Have you used medicinal plants or herbal products for health or well-being in the past year”) alongside detailed use information as indicated in Part three of the survey. Surveys of students who indicated inconsistent answers were scrutinized and, after finding their omission would have no significant impact on our overall findings, omitted from the final statistical analysis presented here.

## Results

### Demographic information

A total of 721 surveys were completed (223 HINU and 498 KU students). In 2012 (the first year student data were available), HINU reported an enrollment of 846 students: 51.7 % female and 48.3 % male [39]. Over 27 % of the HINU student population were surveyed for this research and represented a similar gender distribution (48.6 % female; 51.4 % male). In 2011, KU reported a total enrollment of 25,448 students: 49.8 % female and 50.2 % male [40]. Of 19,222 total KU undergraduates in 2011, nearly 3 % were surveyed for this research and represented a variable, but similar gender distribution (55.6 % female; 44.2 % male). Of undergraduate students enrolled at KU in 2011, 76.8 % identified as White, 5.3 % as Hispanic, 3.8 % as Black, and 3.6 % as Asian [41]. Of students surveyed at KU, 80.2 % identified as White, 3.6 % as Hispanic, 5.7 % as Black, and 5.9 % as Asian, which is comparable to the larger undergraduate student population. Additional demographic data for student enrollment at both Universities were unavailable for determining the representation of age and class in our sample. Table 1 presents a comparison of surveyed students’ demographic information between HINU and KU.

**Table 1:** Demographics of HINU and KU^a^

Demographic Information	HINU	KU	Total
Total Number Cases	223	498	721
Race/ethnicity			
Native American	84.3 %	.4 %	26.5 %
White	.4 %	80.2 %	55.4 %
Asian	-	5.9 %	4.0 %
Black	-	5.7 %	3.9 %
Hispanic/Latino	-	3.6 %	2.5 %
Pacific Islander	-	.2 %	.1 %
2+ Ethnicities	15.2 %	3.6 %	7.2 %
Other	-	.4 %	.3 %
Missing (cases)	-	3	3
Gender			
Male	51.4 %	44.2 %	46.4 %
Female	48.6 %	55.6 %	53.4 %
Transgender	-	.2 %	.1 %
Missing (cases)	11	27	38
Age (in years)			
18-19	57.2 %	79.1 %	72.3 %
20-21	16.7 %	18.0 %	17.6 %
22-23	10.8 %	2.0 %	4.7 %
24-25	4.5 %	.6 %	1.8 %
26-30	5.4 %	-	1.7 %
31-40	3.6 %	.2 %	1.3 %
41+	1.8 %	-	.6 %
Missing (cases)	1	4	5
Class			
Freshman	80.2 %	62.9 %	68.3 %
Sophomore	18.0 %	24.9 %	22.8 %
Junior	1.8 %	8.6 %	6.5 %
Senior	-	3.7 %	2.5 %
Missing (cases)	1	8	9

^a^ Percentage of all students surveyed in each demographic at HINU and KU

### HINU and KU differences

Haskell Indian Nations University students were significantly more likely to rate their health as poor than KU students (p = .000); and significantly more likely to use commercial tobacco products (p = .001). Whereas 73.5 % of KU students rated their health as “good” or “above average,” less than half (46.7 %) of the HINU sample rated their health as “good” or “above average.” Whereas 29.4 % of the HINU sample used commercial tobacco products, only 16.9 % of the KU sample indicated commercial tobacco use. KU students reported significantly higher use rates of prescription medication (50.2 % v. 29.3 % at HINU, p = .000) and OTC medication (62.6 % v. 42.8 % at HINU, p = .000). Many of these health behavior differences persist when comparing past year medicinal plant use between both university student populations.

### HINU and KU past year user differences

Haskell Indian Nations University students reporting past year medicinal plant and herbal product use were significantly older than their KU counterparts (p = .000) (Table 2), despite featuring a higher percentage of underclassmen. The gender makeup of the two groups did not differ significantly (p = .198).

**Table 2:** Demographics of past year users^b^

Demographics of Past Year Users	HINU	KU	Total
Total Number Cases	56	115	171
Percent Past Year Users	25 %	23 %	
Ethnicity			
Native American	80.4 %	-	26.6 %
White	-	86.7 %	58.0 %
Asian	-	1.8 %	1.2 %
Black	-	4.4 %	3.0 %
Hispanic/Latino	-	3.5 %	2.4 %
Pacific Islander	-	.9 %	.6 %
2+ Ethnicities	19.6 %	2.7 %	8.3 %
Other	-	-	-
Missing (cases)	-	2	2
Gender			
Male	56.6 %	45.8 %	49.4 %
Female	43.4 %	54.2 %	50.6 %
Transgender	-	-	-
Missing (cases)	3	8	11
Age (in years)			
18-19	35.7 %	76.1 %	62.7 %
20-21	23.2 %	18.6 %	20.1 %
22-23	12.5 %	3.5 %	6.5 %
24-25	7.1 %	.9 %	3.0 %
26-30	14.3 %	-	4.7 %
31-40	7.1 %	.9 %	3.0 %
41+	-	-	-
Missing (cases)	-	2	2
Class			
Freshman	80.0 %	55.4 %	63.5 %
Sophomore	20.0 %	29.5 %	26.3 %
Junior	-	9.8 %	6.6 %
Senior	-	5.4 %	3.6 %
Missing (cases)	1	3	4

^b^ Percentage of past-year student users in each demographic at HINU and KU

There was no significant difference found between HINU and KU regarding lifetime (p = .951) and past year (p = .384) use of medicinal plants or herbal products. With regard to students indicating past year use, age of first use (p = .284), total money spent on medicinal plant products (p = .910), and whether the student informed their physician of their use (p = .360) were not statistically different between HINU and KU. However, past year HINU users were less likely to report good or above average health (p = .000; 41.1 % v. 79.5 %), use less prescription medication (p = .005; 30.4 % v. 53.1 %) and were almost twice as likely to use commercial tobacco products (p=. 016; 41.8 % v. 23.7 %). While these differences in health, tobacco use, and prescription medication use are also reflected in the overall difference between HINU and KU respondents, a significant difference in OTC medication use was not present in HINU and KU past year users.

Significant differences were also observed with respect to how students learned how to use the medicinal plants and herbal products (Table 3). Most students from both schools learned about medicinal plants from a family member. However, significantly more HINU students learned to use the herbals from an elder or healer, whereas KU students were more likely to learn about medicinal plants from a conventional health practitioner. Also, KU students were more likely to learn about medicinal plants through electronic media such as the internet.

**Table 3:** Where students learn about herbal products^c^

Q4. Where learned to use	Total	HINU	KU	p-value
Total Number Cases	171	56	115	
Family member	64.3 %	60.7 %	66.1 %	.491
Elder or Healer	19.3 %	44.6 %	7.0 %	.000**
Coach/Athletic Trainer	11.7 %	7.1 %	13.9 %	.196
Alternative HP	5.3 %	3.6 %	6.1 %	.489
Conventional HP	11.7 %	3.6 %	15.7 %	.021*
Friend	43.9 %	37.5 %	47.0 %	.242
Electronic media	19.9 %	8.9 %	25.2 %	.012*
Print media	18.1 %	21.4 %	16.5 %	.434
Advertising	12.3 %	5.4 %	15.7 %	.054

^c^ Percentage of past year student users who utilized each information source. Students could select more than one answer

### Reasons for use

University of Kansas students were much more likely to report taking medicinal plants and herbal products for health maintenance than HINU students (90.4 % vs. 60.0 %), and were more likely to use medicinal plants to enhance appearance. HINU students were more likely to report use of medicinal plants for traditional reasons, spiritual reasons, weight loss, sexual purposes, or to treat a mental condition (Table 4). GLM analysis indicated that student choices for using herbal products varied most for treating a chronic condition, treating a physical condition, or treating a mental health condition. F scores from the MANOVA indicate gender, age, and grade contribute most to the variation in choices. Location (KU vs. HINU) was also significant (p = 0.004), as was ethnicity (p = 0.001).

**Table 4:** Indications for medicinal plant use^d^

Indications for Medicinal Plant Use	HINU	KU
Total Number Cases	56	115
Health maintenance	60.0 %	90.4 %
Treat a physical condition	42.2 %	45.7 %
Simply like taking it	48.9 %	29.8 %
Traditional reasons	46.7 %	20.2 %
Treat a chronic condition	20.0 %	29.8 %
Alternative to other medicine	37.8 %	19.1 %
Beauty purposes	8.9 %	25.5 %
Increase energy	22.2 %	16.0 %
Other	31.1 %	10.6 %
Spiritual reasons	33.3 %	3.2 %
Weight loss	13.3 %	5.3 %
Treat a mental condition	17.8 %	2.1 %
Sexual purposes	8.9 %	2.1 %
Muscle gain	2.2 %	1.1 %
Missing (cases)	11	21

^d^ Percentage of past-year student users indicating reasons for use of medicinal plants and herbal products. Students could select more than one answer

* All Cases: All (n = 721); HINU (n = 223); KU (n = 498)

** Valid Cases (Answered ‘yes’ to Q1 & Q2): All (n = 171); HINU (n = 56); KU (n = 115)

Note: of 171 valid cases, only 139 cases (45 HINU; 94 KU) indicated the use of one or more medicinal plants in Part 3 of the survey

There were significant differences between HINU and KU students regarding the sum of medicinal plants used in the past year (Table 5) (p = .003). Haskell Indian Nations University students were more likely to indicate the use of more than one medicinal plant or herbal product.

**Table 5:** Total number of medicinal plants used^e^

Sum of Medicinal Plants Used	Total	HINU	KU
Total Number Cases	171	56	115
1	36.7 %	20.0 %	44.7 %
2	23.7 %	31.1 %	20.2 %
3	12.9 %	13.3 %	12.8 %
4	8.6 %	11.1 %	7.4 %
5	4.3 %	8.9 %	2.1 %
6	2.2 %	2.2 %	2.1 %
7	2.9 %	4.4 %	2.1 %
9	2.2 %	4.4 %	1.1 %
10 or more	6.5 %	6.8 %	5.3 %
Missing (cases)	32	11	21

^e^ Percentage of student users who indicated the number of plants used in the past year

University of Kansas students were more likely to use aloe and Echinacea (Table 6), while HINU students were more likely to use chamomile, ginseng, ginger, gingko, and valerian, among others. For the most part, the two groups showed similar usage rates that did not differ statistically.

**Table 6:** Usage rates of specific medicinal plants and herbal products^f^

Percent of Students Using a Medicinal Plant	HINU	KU
Total Number Cases	56	115
Aloe	73.3 %	88.3 %
Chamomile	42.2 %	27.7 %
Ginseng	37.8 %	27.7 %
Peppermint	22.2 %	24.5 %
Ginger	28.9 %	19.1 %
Echinacea	11.1 %	14.9 %
Garlic	17.8 %	10.6 %
Gingko	17.8 %	9.6 %
Combination Formula	13.3 %	9.6 %
St. John’s wort	11.1 %	8.5 %
Kava	11.1 %	7.4 %
Valerian	15.6 %	5.3 %
Ephedra	6.7 %	8.5 %
Licorice	8.9 %	7.4 %
Milk Thistle	11.1 %	6.4 %
Evening Primrose	6.7 %	6.4 %
Feverfew	4.4 %	6.4 %
Goldenseal	4.4 %	5.3 %
Missing (cases)	11	21

^f^ Percentage of past-year student users indicating use of specific medicinal plants or herbal products

Student respondents were allowed to write-in the names of additional medicinal plants and herbal products that they used (Table 7). The most common write-in among both groups was marijuana. Haskell Indian Nations University students reported a greater number (n = 25 v. n = 13) and wider diversity of write-in products, including cedar, Navajo tea, peyote, bitterroot, horny goat weed, huckleberry tea, Indian tea, sage, saw palmetto, and willow. University of Kansas students added things such as mullein and yerba mate, neither of which is native to North America.

**Table 7:** Additional “write in” medicinal plants and herbal products^g^

Write in Herbal Products	HINU	KU
Total Number Cases	56	115
Marijuana	5	6
Cedar	4	0
Tea tree oil	2	1
Navajo tea	2	0
Peyote	2	0
One occurrence:		
5 HTP	0	1
Cranberry	0	1
Kratom	0	1
Mullein	0	1
Red raspberry leaves	0	1
Yerba Mate	0	1
Angelica root	1	0
Bitterroot/Black Medicine	1	0
Choke Cherry leaf	1	0
Corn pollen	1	0
Horny Goat weed	1	0
Huckleberry tea leaf	1	0
Indian tea	1	0
Sage	1	0
Saw palmetto	1	0
Willow	1	0
Missing (cases)	41	103

^g^ Number of past-year student users indicating use of a medicinal plant or herbal product not listed on the survey

### User and non-user differences

Of the 721 students surveyed, 236 students (32.7 %) indicated lifetime use of medicinal plants or herbal formulas. Of students indicating lifetime use of medicinal plants or herbal formulas, 171 students indicated past year use (23.7 %).

Past year medicinal plant users were older than non-users (p = .001), but gender was not a factor in usage (p = .164). Cigarette smoking was more common among past year users (29.5 %) than among non-users (18.3 %) (p = .002). There were no significant differences in over the counter (OTC) medicine (p = .425) and prescription medication (p = .635) use between past year users and nonusers. Past year users were more likely to report that a physician had inquired about their usage (p = .000), and that they were able to find sufficient information on medicinal plant use (p = .000).

## Discussion

University of Kansas and Haskell Indian Nations University medicinal plant and herbal product use rates (23.7 % past year; 32.7 % lifetime) are higher than the 2007 US adult natural product use rate (17.7 % past year). This is consistent with prior student survey research indicating that college student natural product use rates range from 26.3 % to 79 % [17, 26]. However, KU and HINU students report a lower rate of past year medicinal plant use than previous student surveys, as well as a lower diversity of medicinal plants used (an average of 1.67 compared to 2.2 and 2.7 in previous surveys) [15, 17].

Consistent with previous student surveys, the top sources of medicinal plant knowledge for HINU and KU students are family (64.3 %), friends (43.9 %), and electronic (19.9 %) and print media (18.1 %) (students could select more than one source). In addition, we found that HINU students rely upon another source of knowledge not previously mentioned in student surveys, elder or healer (7 % of all KU past year users; 44.6 % HINU). While Ambrose and Samuels and Newberry et al reported that 3.8 % and 14 % of student users experienced adverse effects, we found only three adverse effects reported (1.2 % of student users): two for ginseng and one for peppermint [15, 28]. In the case of Newberry et al, 11 of the 19 reported adverse effects were from ephedra use—adverse effects not reported by students in this survey [28].

No significant difference was found regarding medicinal plant usage rates between students at the two universities, though there were differences noted in the reasons for usage. We had initially hypothesized that the Native American students at Haskell Indian Nations University would show a higher rate of medicinal plants and herbal product use due to the cultural tendency for tribal family members and elders to pass on traditional medicinal knowledge to younger tribal members, but this was not the case [34, 37]. Native American students who reported medicinal plant use did cite elders or healers as a source of knowledge, and cited traditional and spiritual reasons for using some of the plants. However, this did not translate into more widespread use. In addition, although HINU students are older than KU students, age did not contribute to a greater rate of medicinal plant use. Despite no significant difference in total usage rates, we did find variations in the plants used and reasons for use.

University of Kansas students are more likely to use herbal products for general health maintenance. This may reflect the marketing of many herbal products as substances to be taken regularly for health, similar to multi-vitamin supplements [9]. Under the Dietary Supplement Health and Education Act of 1994, dietary supplements cannot claim to “diagnose, cure, mitigate, treat, or prevent illness”; thus product manufacturers can only make broad health claims regarding their products [42]. Because KU students rely more upon electronic media and advertising for medicinal plant knowledge than HINU students, they may be more exposed to marketing claims, which in turn influences their reasons for using the product. In addition, significantly more KU students rate their health as “good” or “above average” (p = .000), suggesting that their use of dietary supplements may be to maintain their health status.

KU students are more than twice as likely to use herbal products to enhance appearance, perhaps revealing the importance of looks among people at these ages, particularly among women. Those reporting using herbal products to enhance appearance were generally younger and typically female (87 % of KU students reporting use for beauty purposes were female). However, the correlations between age/sex and using herbal products to enhance appearance are not significantly high. It could be that KU students are more influenced by (or exposed to) gender and beauty myths than HINU students. Aloe is the most often cited medicinal plant used to enhance appearance.

Native American students are much more likely to use medicinal plants as alternatives to mainstream medicine. This perhaps indicates a greater acceptance of plants as a source of medicine. Also, the greater usage of herbal medicines for spiritual reasons reflects a Native American cultural connection between medicinal plant use and health and overall well-being [34, 37]. Interestingly, the tendency for Haskell students to have a greater likelihood of using herbal products to enhance mental health may reflect higher stress or anxiety levels among HINU students. Data indicate that Native Americans are more likely to suffer from depression and related mental health conditions [43]. Despite a smaller sample size, HINU students provided a larger number of “write-in” medicinal plants not listed on the survey and indicated use of more medicinal plants that are native to North America. This may also be indicative of exposure to a wider variety of plant medicines and localized knowledge across the lifespan.

The lower self-assessment of health status among Native Americans that we documented have been reported elsewhere, as have higher rates of cigarette smoking and lower utilization of conventional health practitioners and pharmaceuticals. Our results likely reflect historically-entrenched social structures that wrought economic and cultural impoverishment on American Indian reservations. In consequence, American Indians as a whole are significantly more likely to rate their health as below average [44–46], more likely to use tobacco products [47, 48], significantly less likely to see a physician due to cost and inaccessibility [45, 46, 48], and significantly less likely to use physician-prescribed drugs [45]. A lower self-reported health rating and less access to prescription medications did not translate into greater medicinal plant and herbal product usage among HINU students. Although HINU students were older, reported more varied reasons for using medicinal plants, recognized more medicinal plants, and professed greater spiritual and traditional reasons for using the plants as compared to KU students, none of these differences contributed to higher overall medicinal plant use by HINU students.

Similar to previous student surveys, aloe, ginseng, chamomile, ginger, Echinacea, garlic, gingko, combination formulas, St. Johns wort, kava kava, valerian, and ephedra are among the top 15 used herbal products. Peppermint and licorice were also selected by KU and HINU students but not reported in previous student surveys.

Past year medicinal plant and herbal product users in this survey are older than non-users (p = .001), perhaps reflecting the tendency for older individuals to have more health problems or health concerns. Gender was not a factor (p = .164). The use of commercial tobacco products was significantly (p = .002) more prevalent among past-year users than among students who did not use a medicinal plant in the past year. Data from the 2002 National Health Interview Survey and the 1999-2000 National Health and Nutrition Examination Survey indicate that former smokers were much more likely to use dietary supplements [16, 49]. Perkin et al likewise found a significant correlation between dietary supplement use and smoking among college students and concluded that dietary supplement use could be seen as off-setting the unhealthy effects of smoking [17]. It could also be that college students who smoke will become former smokers at some point in their life, which would explain the correlation between smokers in college populations and former smokers in the larger adult population and the use of dietary supplements. Smoking and dietary supplement use may also correlate by way of the same mechanisms that cigarette, alcohol, and/or marijuana use correlate with illicit drug use (e.g., gateway drug hypothesis [50, 51]). That is, students who are willing to smoke cigarettes may also be more willing to experiment with and use medicinal plants and herbal products than their nonsmoking counterparts. While not demonstrated by the data, it is possible that the use of medicinal plants, in addition to tobacco, indicates attempts to self-medicate for anxiety and is a related phenomenon. Lastly, students who used medicinal plants in the past year are also significantly more likely to report that a physician inquired about their use and that they found sufficient information (from any source) about medicinal plant and herbal product use, which likely reflects a greater awareness of medicinal plants among users.

## Conclusions

While we hypothesized that the Native American students from Haskell Indian Nations University would use medicinal plant and herbal products more often than non-Native students from the University of Kansas, this was not the case. However, our results are consistent with previous findings regarding student medicinal plant use, with 23.7 % of students reporting use of medicinal plants in the past year. Despite no significant difference in total usage rates, we did find variations in the plants used and reasons for use. Thus, while rates of medicinal plant use are consistent between Native American and non-Native university students, there are differences in the way that students learn about and use medicinal plants that other research can explore. In addition, significant differences in medicinal plant use rates might be revealed among ethnicities outside of the university student population, including within a wider diversity of ages, education levels, and geographic locations. Additional research could investigate the barriers to medicinal plant use for students and other populations, and how Native American traditional and cultural practices affect medicinal plant use. Further, qualitative research, including interviews and case studies, of different cross-sections of medicinal plant users can reveal details that this survey did not capture, thereby adding explanatory depth to the results reported here and in other surveys of CAM use.

### Limitations

Direct comparison with previous student surveys is somewhat problematic due to variation in herbal product terminology. Newberry et al and Perkin et al measured non-vitamin, non-mineral product/supplement use, which includes products like protein powder and amino acids, green tea, cranberry, and acidophilus (products that were also mentioned in previous herbal use surveys [15, 26] and did not appear in our more medicinal plant-focused survey) [17, 28]. Furthermore, scrutiny of the completed surveys indicated that nearly 20 % of students who indicated past year use of medicinal plants in Part three of the survey had either not indicated past year use in Part two of the survey or changed their answer to reflect past year use. This highlights discrepancies in how the authors and respondents conceived of ‘medicinal plant’ and ‘herbal product’ use. Finally, while herbal retailers were listed as a source of knowledge in previous student surveys, this option was not available on the KU/HINU survey.

## Additional file

Additional file 1:
**Student Medicinal Plant and Herbal Product Survey.**
