# Supplementary material for: Ethnic differences in medicinal plant use among University students: a cross-sectional survey of self-reported medicinal plant use at two Midwest Universities
Source: BMC Complement Altern Med. 2015 Jun 23;15:192. doi: 10.1186/s12906-015-0725-1 (PMC4476180; doi:10.1186/s12906-015-0725-1)
Supplement: Additional file 1: — Student Medicinal Plant and Herbal Product Survey. [file 12906_2015_725_MOESM1_ESM.pdf]

Appendix A: Student Medicinal Plant and Herbal Product Survey

Part I: Demographics:

Ethnicity:

- ◯ White
- ◯ Black
- ◯ American Indian

  Tribal Affiliation:________________________
- ◯ Asian
- ◯ Hispanic or Latino
- ◯ Alaskan Native
- ◯ Pacific Islander
- ◯ Foreign Exchange Student
- ◯ Two or more ethnicities
- ◯ Other

Age:

- ◯ 18-19
- ◯ 20-21
- ◯ 22-23
- ◯ 24-25
- ◯ 26-30
- ◯ 31-40
- ◯ 41+

Grade Level:

- ◯ Freshman
- ◯ Sophmore
- ◯ Junior
- ◯ Senior
- ◯ Graduate

Gender:

- ◯ Male
- ◯ Female
- ◯ Transgendered

Please rate your health on the scale below:

{---}

◯ Good ◯ Below Average ◯ Average ◯ Above Average ◯ Poor

Part II: Medicinal Plant and Herbal Product Use:

1) Have you ever used medicinal plants or herbal products for health or well-being?

◯ Yes ◯ No (Please continue the survey at Question #7 below.)

2) Have you used medicinal plants or herbal products for health or well-being in the past year?

◯ Yes ◯ No

3) At what age did you learn about medicinal plants or herbal products and their use?

- ◯ 0-10 years old
- ◯ 11-16 years old
- ◯ 17-25 years old
- ◯ 26+ years old
- ◯ Not applicable
- ◯ Not sure

4) From whom, or where, have you learned about medicinal plants and their use?
(Please select all that apply.)

- ◯ Family Member
- ◯ Elder or Healer
- ◯ Conventional Health Practitioner (Doctor)
- ◯ Alternative Health Practitioner
- ◯ Coach/Athletic Trainer
- ◯ Friend
- ◯ Electronic Media (E.g., Television, Internet, Radio)
- ◯ Print Media (E.g., Books, Magazines, Newsletters)
- ◯ Advertising

5) On average, how much money do you spend on medicinal plants and herbal products per month?

- ◯ Less than $5
- ◯ $5-$10
- ◯ $10-$25
- ◯ $26-$50
- ◯ $51-100
- ◯ Greater than $100

6) Have you ever told your Doctor about your medicinal plant or herbal product use?

◯ Yes ◯ No ◯ Not Applicable or Don't Remember

7) Has your Doctor ever asked you about medicinal plant or herbal product use?

◯ Yes ◯ No ◯ Not Applicable or Don't Remember

8) Do you take Doctor-prescribed medication?

◯ Yes ◯ No

9) Do you take over-the-counter medicine?

◯ Yes ◯ No

10) Have you found sufficient information on the use of medicinal plants and herbal products?

◯ Yes ◯ No ◯ Haven't looked for information

If you answered yes to Question #2 above, please continue this survey on the other side.
If you have not used medicinal plants or herbal products in the past year, you are finished with this survey. Thank you for your participation.

Part III: If you have used medicinal plants or herbal products in the past year, please indicate your use below:

Medicinal Plant	When you use the plant, how frequently do you use it?				When you use the plant, how long do you use it for?				In what form have you used the plant? (Please check all that apply.)						What have you used this plant for? (Please check all that apply.)														Did the plant work?			Any adverse effects?	
	Daily	Weekly	Monthly	Spradically or as needed	1-6 Days	1-3 Weeks	1-6 Months	More than 6 Months	Dried	Fresh	Tincture (plant extract)	Capsule or Pill	Topically (on the skin)	Infusion (tea)	Health maintainence	Treat a chronic condition	Treat a physical condition	Treat a mental condition	Weight loss	Muscle gain	Increase energy	Beauty purposes	Sexual purposes	I like it	Alternative to other medicine	Traditional reasons	Spiritual reasons	Reason not listed	Effective	Ineffective	Don't Know	Yes	No
Aloe Vera	○	○	○	○	○	○	○	○	○	○	○	○	○	○	○	○	○	○	○	○	○	○	○	○	○	○	○	○	○	○	○	○	○
Chamomile	○	○	○	○	○	○	○	○	○	○	○	○	○	○	○	○	○	○	○	○	○	○	○	○	○	○	○	○	○	○	○	○	○
Combination Formula	○	○	○	○	○	○	○	○	○	○	○	○	○	○	○	○	○	○	○	○	○	○	○	○	○	○	○	○	○	○	○	○	○
Echinacea	○	○	○	○	○	○	○	○	○	○	○	○	○	○	○	○	○	○	○	○	○	○	○	○	○	○	○	○	○	○	○	○	○
Ephedra	○	○	○	○	○	○	○	○	○	○	○	○	○	○	○	○	○	○	○	○	○	○	○	○	○	○	○	○	○	○	○	○	○
Evening Primrose	○	○	○	○	○	○	○	○	○	○	○	○	○	○	○	○	○	○	○	○	○	○	○	○	○	○	○	○	○	○	○	○	○
Feverfew	○	○	○	○	○	○	○	○	○	○	○	○	○	○	○	○	○	○	○	○	○	○	○	○	○	○	○	○	○	○	○	○	○
Garlic (as medicine)	○	○	○	○	○	○	○	○	○	○	○	○	○	○	○	○	○	○	○	○	○	○	○	○	○	○	○	○	○	○	○	○	○
Ginger	○	○	○	○	○	○	○	○	○	○	○	○	○	○	○	○	○	○	○	○	○	○	○	○	○	○	○	○	○	○	○	○	○
Gingko	○	○	○	○	○	○	○	○	○	○	○	○	○	○	○	○	○	○	○	○	○	○	○	○	○	○	○	○	○	○	○	○	○
Ginseng	○	○	○	○	○	○	○	○	○	○	○	○	○	○	○	○	○	○	○	○	○	○	○	○	○	○	○	○	○	○	○	○	○
Goldenseal	○	○	○	○	○	○	○	○	○	○	○	○	○	○	○	○	○	○	○	○	○	○	○	○	○	○	○	○	○	○	○	○	○
Kava Kava	○	○	○	○	○	○	○	○	○	○	○	○	○	○	○	○	○	○	○	○	○	○	○	○	○	○	○	○	○	○	○	○	○
Licorice	○	○	○	○	○	○	○	○	○	○	○	○	○	○	○	○	○	○	○	○	○	○	○	○	○	○	○	○	○	○	○	○	○
Milk Thistle	○	○	○	○	○	○	○	○	○	○	○	○	○	○	○	○	○	○	○	○	○	○	○	○	○	○	○	○	○	○	○	○	○
Peppermint	○	○	○	○	○	○	○	○	○	○	○	○	○	○	○	○	○	○	○	○	○	○	○	○	○	○	○	○	○	○	○	○	○
St. John's Wort	○	○	○	○	○	○	○	○	○	○	○	○	○	○	○	○	○	○	○	○	○	○	○	○	○	○	○	○	○	○	○	○	○
Valerian	○	○	○	○	○	○	○	○	○	○	○	○	○	○	○	○	○	○	○	○	○	○	○	○	○	○	○	○	○	○	○	○	○

Please write in other medicinal plants and herbal formulas used:

________________	○	○	○	○	○	○	○	○	○	○	○	○	○	○	○	○	○	○	○	○	○	○	○	○	○	○	○	○	○	○	○	○	○
________________	○	○	○	○	○	○	○	○	○	○	○	○	○	○	○	○	○	○	○	○	○	○	○	○	○	○	○	○	○	○	○	○	○

Thank you for completing this survey.
